# Lung cancer surgery in HIV‐infected patients: An analysis of postoperative complications and long‐term survival

**DOI:** 10.1111/1759-7714.13519

**Published:** 2020-07-05

**Authors:** Lin Wang, Yongfang Chen, Yifei Wang, Jianjian Liu, Zilu Wen, Hui Chen, Yijun Zhu, Jun Wang, Laiyi Wan, Feng Li, Yanzheng Song

**Affiliations:** ^1^ Department of Thoracic Surgery Shanghai Public Health Clinical Center Shanghai China; ^2^ Department of Pharmacy Shanghai Public Health Clinical Center Shanghai China; ^3^ Department of Ultrasonic Room Shanghai Public Health Clinical Center Shanghai China; ^4^ Department of Experimental Animal Shanghai Public Health Clinical Center Shanghai China; ^5^ Department of Scientific Research Shanghai Public Health Clinical Center Shanghai China

**Keywords:** Human immunodeficiency virus, non‐small cell lung cancer, surgery, surgical outcomes

## Abstract

**Background:**

The purpose of this study was to investigate the risk factors of postoperative complications and reliable prognostic factors of long‐term survival in HIV‐infected patients with non‐small cell lung cancer (NSCLC).

**Methods:**

HIV‐infected patients with NSCLC who underwent surgical treatment were retrospectively studied; a single‐institutional analysis was conducted from November 2011 to August 2018. Pre‐ and postoperative clinical data, including age, gender, smoking history, highly active antiretroviral therapy (HAART), CD4+ T cell count, HIV viral load, cancer histology, clinical and pathological stage (p‐stage), surgical result, Glasgow Prognostic Score (GPS), the Charlson comorbidity index (CCI), survival time and postoperative complications were collected.

**Results:**

A total of 33 HIV‐infected patients with NSCLC were enrolled of which 18 (54.7%) had preoperative comorbidities and postoperative complications were observed in 22 (66.7%) patients. Thirty‐day mortality was not observed in these patients. Median survival time after surgery was 65 months: the MST of p‐stage I patients was 65 months; p‐stage II MST was unestimable; p‐stage III MST was 21 months. Univariate analyses showed that postoperative complications were associated with HIV viral load (*P* = 0.002), CCI (*P* = 0.027), HAART (*P* = 0.028) and CD4+ T cell count (*P* = 0.045). However, multiple logistic regression analysis showed no correlation between HAART and postoperative complications. The p‐stage was an independent prognostic factor for survival time.

**Conclusions:**

In our single‐arm retrospective analysis, the risk factors for postoperative complications in HIV‐infected patients with NSCLC were HIV viral load, CCI and CD4+ T cell counts. The p‐stage was a predictive factor for long‐term survival.

## Introduction

The advent of antiretroviral therapy has led to a decreased mortality from opportunistic infectious diseases associated with acquired immunodeficiency syndrome (AIDS). Similarly, deaths from a wide variety of cancers have been increasing among this population. Lung cancer represents the leading cause of cancer‐related death and a major source of morbidity among persons living with HIV infection in the United States.^1^, ^2^ Although China's national antiretroviral therapy (ART) program began later than those of most developed countries, China might experience HIV‐related cancer epidemics in the ART era. Non‐AIDS‐defining cancers (NADCs) have emerged as a major problem and have contributed to excess mortality among individuals living with HIV: the most prevalent NADCs in China were reported to be Hodgkin's lymphoma (27%), gastrointestinal cancer (16%), liver cancer (14%) and lung cancer (13%), at rates higher than the crude incidence rate of cancer in general populations.^3^

Some factors, such as young age, smoking, persistent HIV RNA viremia, use of intravenous drugs and other comorbidities including chronic obstructive pulmonary disease, recurrent pneumonia and chronic inflammation have been identified as risk factors for lung cancer in these patients.^4^ Cancer stage, tumor characteristics and patient factors influence the outcome of lung cancer treatments. Some studies suggest that immunosuppression may not play a role in lung cancer development. Other studies show that nadir in CD4 + T cell count and length of immunosuppression may be important to take into consideration.^5^ The role of persistent HIV viral load in the pathogenesis and development of lung cancer remains undefined.^6^ Young age, male sex and extensive smoking history seem important to take into consideration.^7^

Surgical resection is regarded as a major component of lung cancer treatment for stage I–IIIA disease and can be curative in some circumstances.^8^, ^9^, ^10^ However, the published studies of surgical treatment mostly came from HIV‐infected lung cancer patients in Western counties, few studies have been reported from Asian populations, and they have crucially lacked detailed clinical characteristics. In the only report from Asian countries other than Japan, only 12 lung cancer patients with HIV or AIDS were included and their detailed clinical characteristics were not documented (TREAT Asia HIV Observational Database).^11^ We therefore studied the clinical characteristics and surgery outcomes of Chinese lung cancer patients infected with HIV in our institution. This study drew upon an eight‐year retrospective cohort at Shanghai Public Health Clinical Center to focus retrospectively on clinical data among HIV‐infected patients with non‐small cell lung cancer (NSCLC). We examined the relevance of HIV disease features (CD4+ T cell count, HIV viral load and use of antiretroviral treatment) for survival time and aimed to elucidate the risk factors for postoperative complication in this Chinese single‐institutional cohort.

## Methods

## Patient inclusion criteria

This study included HIV‐infected patients with NSCLC at Shanghai Public Health Clinical Center (Department of Thoracic Surgery) from November 2011 to August 2018.

Criteria for patient inclusion were as follows: (i) Not limited by age or gender; (ii) met the diagnostic criteria for adult HIV/AIDS developed by the Centers for Disease Control and Prevention (CDC) in 1993 and a whole‐blood confirmatory test conducted by the local CDC laboratory was positive for anti‐HIV‐1 antibody; (iii) compliance with the TNM staging of lung cancer in the eighth edition interpretation of the International Association for the study of Lung Cancer (IASLC); (iv) postoperative pathological diagnosis of NSCLC; and (v) the presence of clinical stage I–IIIA of lung cancer.

Clinical characteristics were retrospectively collected, including age, gender, preoperative comorbidity, preoperative blood test results (CD4 + T cell count, HIV viral load, C‐reactive protein (CRP) and albumin levels), smoking history, HAART, tumor histology, clinical and pathological stage (p‐stage), operative procedure, operative time, postoperative complication, survival time and cause of death. The preoperative examination for cancer staging included computed tomography (CT) of the chest and upper abdomen, CT or magnetic resonance imaging (MRI) of the brain and bone scintigraphy or ^18^F‐fluorodeoxy glucose‐positron emission tomography. Although mediastinoscopy was not performed in all patients, bronchoscopy was performed in some cases.

The CCI was scored according to 19 preoperative comorbidities; the method was consistent with that of Charlson *et al*.^12^ The GPS was defined as follows: score 0, albumin level ≥ 3.5 g/dL and CRP level <0.5 mg/dL; score 1, albumin level <3.5 g/dL or CRP level ≥ 0.5 mg/dL; score 2, albumin level <3.5 g/dL and CRP ≥0.5 mg/dL. This scoring method was similar to that of McMillan *et al*.^13^ Postoperative pulmonary complications were defined as follows: Pulmonary fistula, prolonged air leakage over seven postoperative days or the need for reoperation or pleurodesis; Postoperative pneumonia, new infiltration on chest roentgenogram (X‐P) or CT scan which needed antibiotic therapy; respiratory failure, management with a ventilator over two postoperative days, or the need for reintubation or tracheotomy. The overall survival time was calculated from the date of surgery to the time of death or the last follow‐up. The follow‐up was until 18 September 2019. This study was approved by the ethics committee of the Shanghai Public Health Clinical Center (Approval No. [2018] Y038).

Statistical analysis was performed using SPSS 22.0 (IBM SPSS Statistics for Windows; IBM Corp., Armonk, NY). The risk factors for postoperative complications were assessed using uni‐ and multivariate logistic regression analyses. The p‐stage was analyzed as a categorical variable. Survival curves were calculated via the Kaplan‐Meier method; differences in survival were assessed using the log‐rank test. Univariate analyses for long‐term survival were calculated via the Cox proportional hazard model. A *P*‐value <0.05 was considered statistically significant. Only variables that could significantly (*P* < 0.05) predict the dependent variable in the univariate logistic regression were included in the multivariate logistic regression and survival analysis. The method of forward stepwise (likelihood ratio) was applied in the multivariate logistic regression analysis and the multivariate survival analysis to develop the best predictive equation model.

## Results

**Figure 1:**
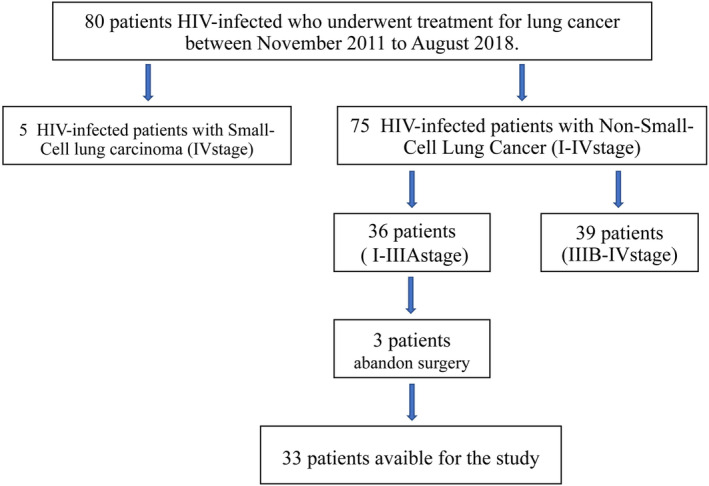
Flow chart of the patients eligible for this study.

A total of 75 HIV‐infected patients with NSCLC at Shanghai Public Health Clinical Center (Department of Thoracic Surgery) from November 2011 to August 2018 were included in the study. Of these, 52% (39/75) patients were diagnosed at advanced stages (IIIB–IV), 48%(36/75) patients were diagnosed at early stages (I–IIIA), but three patients were excluded due to surgery being abandoned. Finally, 33 patients (median age 56 years, 27 men) were included in the analysis (Fig 1). The demographics and clinical characteristics of these patients are presented in Table 1. At diagnosis, mean serum CD4 + T cell count was 343.24 ± 148.84/μL (range 82–641/mm^3^).HIV virus was detected in 23 of the 33 patients and the highest preoperative HIV viral load was 3.51E + 05 copy/mL. In total, 20 patients had been treated with HAART before lung cancer was diagnosed, and 13 patients were diagnosed with lung cancer and HIV infection, and did not receive HAART. The mean size of tumor was 3.4 cm (range 1.1–8.9 cm). The histological type of lung cancer was adenocarcinoma in 20 patients, squamous cell carcinoma in eight patients, and others included NSCLC in five patients. The clinical stage was IA in 15 patients, IB in six patients, IIA in one patient, IIB in seven patients, and IIIA in four patients. However, the pathological stage was slightly different to the clinical stage; pathological stage was IA in 19 patients, IB in five patients, IIA in one patient, IIB in three patients, IIIA in four patients, and IV in one patient. Clinical lymph node stage and pathological lymph node stage were also compared, and the former (N0/N1/N2/NX: 29/2/2/0) was slightly different from the latter (N0/N1/N2/NX: 23/3/3/4). A total of eight patients (24.3%) underwent segmentectomy, 23 (69.7%) underwent lobectomy, and two (6%) bilobectomy. Thoracotomy was performed in 23 patients (69.7%), and VATS in 10 patients (30.3%). Among all 33 patients who underwent surgery, 30 patients were simultaneously taking antiretroviral and chemotherapy agents; and one patient was simultaneously taking antiretroviral and targeted drug therapy. The other two patients gave up postoperative chemotherapy. Two patients with postoperative recurrence were given simultaneous antiretroviral and targeted drug therapy. There were 10 patients who died from recurrence and metastasis of lung cancer. The average survival time was 34.3 months. Another three patients with relapse are still receiving targeted drug treatment, and the current average survival time is 26.7 months (Table 3). The current mean observation time of all patients is 35.3 months (range 13 to 94 months). Among the four patients who underwent gene testing, two patients harbored an *EGFR* mutation, one had an *ERBB2* mutation, and one patient had a *KRAS*/*PIK3CA*/*RET* mutation (Table 1).

**Figure 2:**
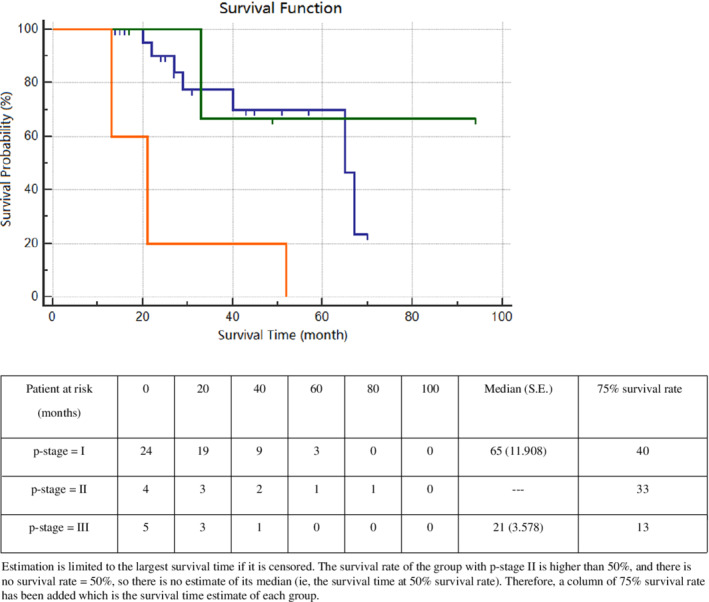
Comparisons of overall survival curves between p‐stage I, II and III patients (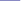) 1, (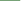) 2, (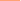) 3.

**Table 1:** Demographic and clinical characteristics of the 33 patients

Demographics	
Mean age, years	30–77 (median 56)
Male/female	27/6
Body mass index	18.8–30.9 (median 22.8)
Brinkman index0/<600/>600	23/3/7
HIV RNA (copy/mL) 0/<40/≥40	10/6/17
CD4 cell count (cell/μL) ≤ 200/200–410/≥410	5/18/10
HAART (month) Yes/No	20/13
Preoperative comorbidity (Yes) (n%)	18 (54.5)
Clinical tumor size (cm)	1.1–8.9 (median 3.4)
Clinical lymph node stage (N0/N1/N2)	29/2/2
Clinical stage (IA/IB/IIA/IIB/IIIA)	15/6/1/7/4
Procedure, n (%)	
Partial resection	8 (24.3)
Lobectomy	23 (69.7)
Bilobectomy	2 (6)
Surgical approach, n (%)	
Thoracotomy	23 (69.7)
VATS	10 (30.3)
Operation time (minutes)	80–360 (median201.4)
Bleeding (mL)	100–600 (median257.7)
Pathological tumor size (cm)	0.7–7 (median2.8)
Pathological lymph node stage (N0/N1/N2/Nx)	23/3/3/4
Pathological stage (IA/IB/IIA/IIB/IIIA/IV)	19/5/1/3/4/1
Pathological type (Ad/Sq/others)	20/8/5
Drainage time (days)	8–33 (median 15.9)
Postoperative drainage (mL)	980–6930 (median 3158.4)
Postoperative adjuvant therapy for pathological stage IIIA and IV (chemotherapy/targeted drug therapy)	4/1
Observation time (months)	13–94 (median35.3)

Ad, adenocarcinoma; VATS, video‐assisted thoracoscopic surgery; Sq, squamous cell carcinoma.

The prevalence of the comorbidity in accordance with the CCI score and the GPS is described in Table 2. The CCI was greater than or equal to eight in 26 (78.7%) of the patients, and 25 (75.8%) had GPS equal to zero. The most common comorbidities were congestive heart failure (six cases, 18.2%) and chronic pulmonary disease (six cases, 18.2%). A total of 22 (66.7%) patients developed postoperative complications and seven of them developed multiple complications (Table 3). During the time of observation, 20 (60.6%) patients were alive without cancer, three patients were alive with cancer, 10 patients had died of cancer; and none of the patients had died due to surgery‐related causes. A 30‐day mortality was observed in none of the patients. We defined patients who had relapsed, metastasized or died at the last follow‐up time as cases of cancer recurrence and designated them as the Cancer Group; patients with no recurrence were designated as the Cure Group. The variables of BI, operative time and p‐stage were selected into the multivariate survival analysis via forward stepwise (likelihood ratio). A best model is reported in Table 4, in which the combination of BI and p‐stage formed the best model. The −2 log likelihood was 56.773, Chi‐square was 19.745, *P* < 0.001. According to the uni‐ and multivariate logistic regression analyses, p‐stage was an independent significant risk factor for cancer return; ie, alive with cancer or died of cancer (odds ratio 1.281–4.818, *P =* 0.016; Table 4). The Kaplan‐Meier curves stratified based on p‐stage are shown in Figure 2. Median survival time was 65 months: for p‐stage I patients MST was 65 months; for p‐stage II MST was unestimable; for p‐stage III it was 21 months; and for p‐stage I/II it was 67 months. The average follow‐up time of those three groups were: 35.5, 48.3, and 26.8 months, respectively. The variables of BI, HIV RNA, Haart, CCI, and CD4 cell count were selected into the multivariate logistic regression analysis via forward stepwise (likelihood ratio). A best model was reported in Table 5 in which the combination of HIV RNA, CCI, and CD4 cell count formed the best model. Its Nagelkerke R2 was 0.63 and the test of the goodness‐of‐fit by the Hosmer‐Lemeshow test showed an appropriate fit with a *P*‐value of 0.635. It should be noted that in the optimal model, all three variables can independently predict the postoperative complications of patients. According to the uni‐ and multivariate logistic regression analyses, independent significant risk factors for postoperative complications were HIV viral load (odds ratio 1.008–92.546, *P =* 0.049), CCI (odds ratio 1.161–199.791, *P =* 0.038), and CD4+ T cell count (odds ratio 0.008–0.949, *P* = 0.045) (Table 5).

**Table 2:** Prevalence of comorbidity scored via the CCI and the GPS

Score	Comorbidity	n (%)	Total score of CCI	n (%)
1	Myocardial infarction	1 (3)	6	2 (6.1)
1	Congestive heart failure	6 (18.2)	7	5 (15.2)
1	Peripheral vascular disease	0	8	8 (24.2)
1	Cerebrovascular disease	1 (3)	9	9 (27.3)
1	Dementia	0	10	5 (15.2)
1	Connective tissue disease	0	11	1 (3)
1	Peptic ulcer disease	0	12	1 (3)
1	Chronic liver disease	0	13	1 (3)
1	Chronic pulmonary disease	6 (18.2)	16	1 (3)
1	Diabetes	1 (3)	Total	33 (100)
2	Hemiplegia	1 (3)		
2	Moderate to severe kidney disease	0		
2	Diabetes with organ damage	0		
2	Solid tumor (within 5 years)	1 (3)		
2	Leukemia	0		
2	Lymphoma	1 (3)		
3	Moderate to severe liver disease	1 (3)		
6	Metastatic solid tumor	0		
6	AIDS	33(100)		
Score	Preoperative state	n (%)	Total score of GPS	n (%)
0	Albumin >3.5 g/dL and CRP <0.5 mg/dL	25 (75.8)	0	25 (75.8)
1	Albumin <3.5 g/dL and CRP <0.5 mg/dL	0	1	0
1	Albumin >3.5 g/dL and CRP >0.5 mg/dL	8 (24.2)	1	8 (24.2)
2	Albumin <3.5 g/dL and CRP >0.5 mg/dL	0	2	0
			Total	33 (100)

AIDS, acquired immune deficiency syndrome; CCI, Charlson comorbidity index; CRP, C‐reactive protein; GPS, Glasgow Prognostic Score.

**Table 3:** Prevalence of postoperative complications, surgery‐related deaths and long‐term outcome

	n (%)	p‐stage	n (%)	Mean follow‐up time (months)
Postoperative complications				
Postoperative pneumonia	19			
Arrhythmia	1			
Respiratory failure	1			
Atelectasis	6			
Postoperative bleeding	1			
Pulmonary fistula	1			
Total	29			
Long‐term outcome				
Alive without cancer	20	I stage	17	34.2
		II stage	3	53.3
		III stage	0	
Alive with cancer	3	I stage	2	33.5
		II stage	0	
		III stage	1	13
Died of cancer	10	I stage	5	40.6
		II stage	1	33
		III/V stage	4	26.8
Total	33			

Note: Postoperative complications occurred in 22 cases, and seven cases had multiple complications.

There were 29 kinds of complications.

**Table 4:** Uni‐ and multivariate analyses with Cox proportional hazards model for overall survival in 33 HIV‐infected patients with non‐small cell lung cancer (NSCLC) who underwent surgery

			Univariate analysis			Multivariate analysis	
Variables	Cured group (20 cases)	Cancer group (13 cases)	Wald	Odds ratio (95% CI)	*P*‐value	Odds ratio (95% CI)	*P*‐value
Age (years)	30–69 (median 56)	41–77 (median 57)	0.008	0.940–1.058	0.927		
Gender (Male/Female)	16/4	11/2	0.119	0.2168–3.495	0.730		
BI (<600/≥600)	17/3	8/5	4.599	1.124–13.548	0.032	0.915–11.57	0.068
Body mass index	18.83–30.85 (median 23.25)	19.03–24.34 (median 22.15)	0.804	0.658–1.168	0.370		
HIV RNA (0/1)	6/14	4/9	0.135	0.245–2.618	0.713		
Diabetes mellitus (Yes/No)	1/19	0/13	0.219	0–18 736.18	0.640		
Haart (Yes/No)	10/10	10/3	2.068	0.708–9.469	0.150		
Comorbidity (Yes/No)	8/12	8/5	0.755	0.535–5.065	0.385		
GPS (1 /0)	5/15	4/9	1.045	0.090–2.128	0.307		
CCI (≤7/≥8)	5/15	2/11	0.729	0.426–8.747	0.393		
Procedure (radical/limited)	15/5	10/3	0.113	0.213–2.987	0.737		
Surgical approach (VATS/thoracotomy)	8/12	2/11	0.293	0.143–3.015	0.588		
Operative time (minutes)	80–350 (median 180)	120–360 (median 180)	2.782	0.982–1.001	0.095		
Bleeding (mL)	100–500 (median 200)	100–600 (median 200)	0.267	0.996–1.003	0.606		
Pathology (non‐Ad/Ad)	7/13	6/7	< 0.001	0.3819–3.065	0.984		
p‐stage (I/II/III)	17/3/0	7/1/5	7.245	1.281–4.818	0.007	1.173–4.712	0.016
CD4 cell count (<200/200–550/>500)	2/15/3	3/8/2	0.634	0.215–1.914	0.426		

Ad, adenocarcinoma; BI, Brinkman index; CCI, Charlson comorbidity index; CI, confidence interval; GPS, Glasgow Prognostic Score; p‐stage, pathological stage; radical, radical lung resection; VATS, video‐assisted thoracoscopic surgery.

**Table 5:** Uni‐ and multivariate logistic regression analyses of variables associated with postoperative complications

			Univariate analysis		Multivariate analysis	
Variables	Complicated group (22 cases)	Noncomplicated group (11 cases)	Odds ratio (95% CI)	*P*‐value	Odds ratio (95% CI) *P*‐value	
Age (years)	44‐69(median59)	30‐77(median53)	0.978–1.149	0.153		
Gender (Male/Female)	18/4	9/2	0.153–6.531	1.000		
BI (0/<600/>600)	13/2/7	10/1/0	0.802–24.984	0.088		
Body mass index	20.48–26.37 (median23.25)	18.83–30.85 (median21.97)	0.903–1.951	0.15		
HIV RNA (0/1)	9/19	7/4	1.966–62.498	0.002	1.008–92.546	0.049
Diabetes mellitus (yes/no)	1/21	0/11		1.000		
Haart (Yes/No)	10/12	10/1	1.303–110.525	0.028		
Comorbidity (Yes/No)	11/11	5/6	0.281–5.124	0.806		
GPS (1 /0)	4/18	5/6	0.053–1.330	0.107		
CCI (≤7/≥8)	2/20	5/6	1.276–54.423	0.027	1.161–199.791	0.038
Procedure (radical/limited)	18/4	7/4	0.5–13.229	0.258		
Surgical approach (VATS/thoracotomy)	6/16	4/7	0.14–3.079	0.593		
Operative time (minutes)	80–360 (median180)	120–350 (median210)	0.985–1.006	0.412		
Bleeding (mL)	100–600 (median200)	100–600 (median300)	0.992–1.002	0.193		
Pathology (non‐Ad/Ad)	9/13	4/7	0.185–3.676	0.801		
p‐stage (I/II/III)	14/3/5	10/1/0	0.680–25.972	0.122		
CD4 cell count (<200/200–500/>500)	5/16/1	0/7/4	0.009–0.733	0.025	0.008–0.949	0.045

Ad, adenocarcinoma; BI, Brinkman index; CCI, Charlson comorbidity index; CI, confidence interval; GPS, Glasgow Prognostic Score; p‐stage, pathological stage; radical, radical lung resection; VATS, video‐assisted thoracoscopic surgery.

## Discussion

With the increasing number of non‐AIDS defining malignancies, cancer screening has become an important component of health maintenance in HIV clinical practice. With increases in lung cancer screening, especially in low‐dose CT screening, the observed prevalence of localized cancers is also likely to increase in HIV‐infected patients.^14^, ^15^ Surgical resection for early‐stage lung cancer is potentially curative. It is time to establish the safety of lung cancer surgery in this group and decrease the existing disparities in cancer treatment in China. Using prospectively collected surgical outcomes data, we found that a lower CD4+ T cell count and higher HIV viral load were associated with higher complication rates post operation. The use of HAART before the operative procedure resulted in no appreciable decrease in complication occurrence. Additionally, immune status at surgery was irrelevant to long‐term survival, which was only associated with the p‐stage of lung cancer in our cohort. These findings further validate prior recommendations advocating surgery for HIV‐infected patients regardless of immune status.^15^

Previous data on postoperative outcomes for HIV‐infected patients specific to lung cancer surgery are limited. In our study, we found that HIV‐infected patients with NSCLC had higher rates of some post‐operative complications. The most common complications were postoperative pneumonia and atelectasis, which were thought to be associated with decreased immune status.^16^, ^17^, ^18^ Treatment with HAART has been indicated as being protective against opportunistic and other infections in patients not undergoing surgery. This was found to be the case in our study. Our results of univariate analysis suggested a history of HAART use 30 days before surgery was associated with a lower incidence of postoperative complications than when there was no history of HAART use. However, there was no significant difference by multivariate analysis.

Previous studies have suggested that CCI and GPS can be used as prognostic markers to predict overall survival in patients with NSCLC.^19^, ^20^, ^21^, ^22^ The GPS and p‐stage were significant prognostic factors in 98 patients aged 80 years and older who had clinical stage I lung cancer.^20^ In another study including 337 lung cancer patients older than 80 years, uni‐ and multivariate analyses showed that p‐stage, CCI, GPS were independent prognostic factors for overall survival.^22^ However, neither the CCI nor the GPS were correlated with cancer‐related deaths in our study. All of the deaths were due to lung cancer recurrence and metastasis. This may be due to the small patient population in our single‐center study. Additionally, some patients were not observed for a significant duration. Further study is needed to establish whether CCI and GPS can be regarded as a reliable predictive markers of survival time for HIV‐infected patients with NSCLC.

Common opportunistic infections in HIV‐infected patients include pneumocystis pneumonia, pulmonary tuberculosis, nontuberculous mycobacterial infection, pulmonary cryptococcus infection, and cytomegalovirus infection. In these patients with recurrent opportunistic pulmonary infection, adhesion of the pleural and mediastinal lymph nodes is frequently found, In our case series, 10 cases of pleural and mediastinal lymph node adhesions were found intraoperatively. Tracing the medical histories revealed that they all had previous opportunistic lung infections. These included eight cases of tuberculosis infection and two cases of pneumocystis pneumonia. Pleural and mediastinal lymph node adhesion is a contraindication for thoracoscopic surgery and resulted in a high proportion of our patients with early stage lung cancer undergoing thoracotomy.

Historically, it has been agreed that HIV‐positive individuals tend to have more advanced cancer stage at diagnosis and that this might contribute to poorer outcomes. Other studies have also shown that HIV‐infection patients with lung cancer have a worse prognosis than those in the general lung cancer population.^23^, ^24^, ^25^, ^26^ However, this understanding of poorer outcomes in HIV‐related lung cancer has been recently challenged. Another study based on the Surveillance Epidemiology and End Results (SEER) cancer registry from 2000 and 2005 evaluated 322 persons with HIV‐associated NSCLC compared to 71 976 HIV‐negative controls with lung cancer and found no difference in stage at lung cancer diagnosis. HIV‐infected patients who underwent surgical resection had similar survival when compared to HIV‐negative counterparts.^27^ In this study, it is p‐stage, not HIV‐related clinical indicators such as HIV viral load and CD4 + T cells, that was found to be associated with survival time. The mean survival time for HIV‐infected patients with stage I/II NSCLC undergoing surgical resection was 67 months, which was better than the report from Hooker *et al*.^7^ (20.4 months of median time for 22 HIV‐infected patients with stage I–III after operation) and similar to results of a population‐based study (median survival for HIV‐infected patients with stage I or II NSCLC undergoing surgical resection was 50 months).^27^

This study has several obvious limitations, including a small sample size and the absence of a comparative analysis between HIV‐infected patients and those without HIV infection. Nevertheless, using data from our Chinese single‐institutional cohorts, we found that the risk factors for postoperative complications were HIV viral load, CCI and CD4 + T cell count, and that p‐stage was the predictive factor for long‐term survival in our single‐arm retrospective analysis. Despite the lower smoking prevalence found in this study, smoking cessation strategies are strongly recommended due to the potential contribution of smoking to lung cancer pathogenesis. In our study, we treated HIV‐infected patients with NSCLC according to the treatment guide for lung cancer in the general population. Such patients were maintained on combination antiretroviral treatment during cancer treatment. Considering the drug‐drug interactions and overlapping toxicity during the concomitant administration of chemotherapy with antiretroviral treatment, it is crucial to have multidisciplinary collaboration among thoracic surgeons, pulmonary specialists, radiologists, and medical and radiotherapy oncologists to obtain the optimal treatment in these patients.

## Disclosure

The authors declare no conflicts of interest.

## References

[1] Aberle DR , Adams AM , Berg CD *et al* Reduced lung‐cancer mortality with low‐dose computed tomographic screening. N Engl J Med 2011; 365: 395–409. 21714641 10.1056/NEJMoa1102873 PMC4356534

[2] Biggar RJ , Engels EA , Ly S *et al* Survival after cancer diagnosis in persons with AIDS. J Acquir Immune Defic Syndr 2005; 39: 293–9. 15980688 10.1097/01.qai.0000164033.02947.e3

[3] Brock MV , Hooker CM , Engels EA *et al* Delayed diagnosis and elevated mortality in an urban population with HIV and lung cancer: Implications for patient care. J Acquir Immune Defic Syndr 2006; 43: 47–55. 16936558 10.1097/01.qai.0000232260.95288.93

[4] Cadranel J , Garfield D , Lavole A , Wislez M , Milleron B , Mayaud C . Lung cancer in HIV infected patients: Facts, questions and challenges. Thorax 2006; 61: 1000–8. 17071836 10.1136/thx.2005.052373 PMC2121163

[5] Charlson ME , Pompei P , Ales KL , MacKenzie CR . A new method of classifying prognostic comorbidity in longitudinal studies: Development and validation. J Chronic Dis 1987; 40: 373–83. 3558716 10.1016/0021-9681(87)90171-8

[6] Church TR , Black WC , Aberle DR *et al* Results of initial low‐dose computed tomographic screening for lung cancer. N Engl J Med 2013; 368: 1980–91. 23697514 10.1056/NEJMoa1209120 PMC3762603

[7] Clifford GM , Polesel J , Rickenbach M *et al* Cancer risk in the Swiss HIV cohort study: Associations with immunodeficiency, smoking, and highly active antiretroviral therapy. J Natl Cancer Inst 2005; 97: 425–32. 15770006 10.1093/jnci/dji072

[8] Clifford GM , Lise M , Franceschi S *et al* Lung cancer in the Swiss HIV cohort study: Role of smoking, immunodeficiency and pulmonary infection. Br J Cancer 2012; 106: 447–52. 22240797 10.1038/bjc.2011.558 PMC3273350

[9] Coghill AE , Pfeiffer RM , Shiels MS , Engels EA . Excess mortality among HIV‐infected individuals with cancer in the United States. Cancer Epidemiol Biomarkers Prev 2017; 26: 1027–33. 28619832 10.1158/1055-9965.EPI-16-0964 PMC5500417

[10] Engels EA , Biggar RJ , Hall HI *et al* Cancer risk in people infected with human immunodeficiency virus in the United States. Int J Cancer 2008; 123: 187–94. 18435450 10.1002/ijc.23487

[11] Engsig FN , Kronborg G , Larsen CS *et al* Lung cancer in HIV patients and their parents: A Danish cohort study. BMC Cancer 2011; 11: 272. 21702995 10.1186/1471-2407-11-272 PMC3135571

[12] Ferreira MP , Coghill AE , Chaves CB *et al* Outcomes of cervical cancer among HIV‐infected and HIV‐uninfected women treated at the Brazilian National Institute of cancer. Aids 2017; 31: 523–31. 28060014 10.1097/QAD.0000000000001367 PMC5263104

[13] Frisch M , Biggar RJ , Engels EA , Goedert JJ . Association of cancer with AIDS‐related immunosuppression in adults. Jama 2001; 285: 1736–45. 11277828 10.1001/jama.285.13.1736

[14] Hino H , Karasaki T , Yoshida Y *et al* Risk factors for postoperative complications and long‐term survival in lung cancer patients older than 80 years. Eur J Cardiothorac Surg 2018; 53: 980–6. 29272371 10.1093/ejcts/ezx437

[15] Hooker CM , Meguid RA , Hulbert A *et al* Human immunodeficiency virus infection as a prognostic factor in surgical patients with non‐small cell lung cancer. Ann Thorac Surg 2012; 93: 405–12. 22269705 10.1016/j.athoracsur.2011.11.012 PMC3298359

[16] Karp J , Profeta G , Marantz PR , Karpel JP . Lung cancer in patients with immunodeficiency syndrome. Chest 1993; 103: 410–3. 8432128 10.1378/chest.103.2.410

[17] Lealdini V , Trufelli DC , Da SF *et al* Applicability of modified Glasgow prognostic score in the assessment of elderly patients with cancer: A pilot study. J Geriatr Oncol 2015; 6: 479–83. 26439755 10.1016/j.jgo.2015.09.001

[18] McMillan DC , Elahi MM , Sattar N , Angerson WJ , Johnstone J , McArdle CS . Measurement of the systemic inflammatory response predicts cancer‐specific and non‐cancer survival in patients with cancer. Nutr Cancer 2001; 41: 64–9. 12094630 10.1080/01635581.2001.9680613

[19] Miura N , Kohno M , Ito K *et al* Lung cancer surgery in patients aged 80 years or older: An analysis of risk factors, morbidity, and mortality. Gen Thorac Cardiovasc Surg 2015; 63: 401–5. 25868520 10.1007/s11748-015-0546-7

[20] Miyazaki T , Yamasaki N , Tsuchiya T *et al* Ratio of C‐reactive protein to albumin is a prognostic factor for operable non‐small‐cell lung cancer in elderly patients. Surg Today 2017; 47: 836–43. 27853867 10.1007/s00595-016-1448-8

[21] Pakkala S , Chen Z , Rimland D *et al* Human immunodeficiency virus‐associated lung cancer in the era of highly active antiretroviral therapy. Cancer 2012; 118: 164–72. 21713759 10.1002/cncr.26242 PMC3184336

[22] Petoumenos K , Hui E , Kumarasamy N *et al* Cancers in the TREAT Asia HIV observational database (TAHOD): A retrospective analysis of risk factors. J Int AIDS Soc 2010; 13: 51. 21143940 10.1186/1758-2652-13-51 PMC3019126

[23] Rengan R , Mitra N , Liao K , Armstrong K , Vachani A . Effect of HIV on survival in patients with non‐small‐cell lung cancer in the era of highly active antiretroviral therapy: A population‐based study. Lancet Oncol 2012; 13: 1203–9. 23164952 10.1016/S1470-2045(12)70466-7

[24] Robbins HA , Pfeiffer RM , Shiels MS , Li J , Hall HI , Engels EA . Excess cancers among HIV‐infected people in the United States. J Natl Cancer Inst 2015; 6 107:4. 10.1093/jnci/dju503 PMC4334816 25663691

[25] Sigel K , Makinson A , Thaler J . Lung cancer in persons with HIV. Curr Opin HIV AIDS 2017; 12: 31–8. 27607596 10.1097/COH.0000000000000326 PMC5241551

[26] Simard EP , Pfeiffer RM , Engels EA . Cumulative incidence of cancer among individuals with acquired immunodeficiency syndrome in the United States. Cancer 2011; 117: 1089–96. 20960504 10.1002/cncr.25547 PMC3052856

[27] Yang J , Su S , Zhao H *et al* Prevalence and mortality of cancer among HIV‐infected inpatients in Beijing. China. BMC Infect Dis 2016; 16: 82. 26883427 10.1186/s12879-016-1416-3 PMC4756453

